# Bracing for the next wave: A critical incident study of frontline decision‐making, adaptation and learning in ambulance care during COVID‐19

**DOI:** 10.1111/jan.16340

**Published:** 2024-07-17

**Authors:** Ann‐Therese Hedqvist, Mats Holmberg, Petronella Bjurling‐Sjöberg, Mirjam Ekstedt

**Affiliations:** ^1^ Department of Health and Caring Sciences Linnaeus University Växjö Sweden; ^2^ Ambulance Service, Region Kalmar Västervik Sweden; ^3^ Department of Ambulance Service Region Sörmland Sweden; ^4^ Centre for Clinical Research Uppsala University Uppsala Sweden; ^5^ Department of Public Health and Caring Sciences Uppsala University Uppsala Sweden; ^6^ Department of Patient Safety Region Sörmland Sweden; ^7^ Department of Learning, Informatics, Management and Ethics, LIME Karolinska Institutet Stockholm Sweden

**Keywords:** ambulance care, COVID‐19, critical incident technique, decision‐making, interpretive description, resilience

## Abstract

**Aim:**

To explore frontline decision‐making, adaptation, and learning in ambulance care during the evolving COVID‐19 pandemic.

**Design:**

Descriptive and interpretative qualitative study.

**Methods:**

Twenty‐eight registered nurses from the Swedish ambulance services described 56 critical incidents during the COVID‐19 pandemic through free‐text questionnaires. The material was analysed using the Critical Incident Technique and Interpretive Description through the lens of potential for resilient performance.

**Results:**

The findings were synthesized into four themes: ‘Navigating uncharted waters under never‐ending pressure’, ‘Balancing on the brink of an abyss’, ‘Sacrificing the few to save the many’ and ‘Bracing for the next wave’. Frontline decision‐making during a pandemic contribute to ethical dilemmas while necessitating difficult prioritizations to adapt and respond to limited resources. Learning was manifested through effective information sharing and the identification of successful adaptations as compared to maladaptations.

**Conclusions:**

During pandemics or under other extreme conditions, decisions must be made promptly, even amidst emerging chaos, potentially necessitating the use of untested methods and ad‐hoc solutions due to initial lack of knowledge and guidelines. Within ambulance care, dynamic leadership becomes imperative, combining autonomous frontline decision‐making with support from management. Strengthening ethical competence and fostering ethical discourse may enhance confidence in decision‐making, particularly under ethically challenging circumstances.

**Impact:**

Performance under extreme conditions can elevate the risk of suboptimal decision‐making and adverse outcomes, with older adults being especially vulnerable. Thus, requiring targeted decision support and interventions. Enhancing patient safety in ambulance care during such conditions demands active participation and governance from management, along with decision support and guidelines. Vertical communication and collaboration between management and frontline professionals are essential to ensure that critical information, guidelines, and resources are effectively disseminated and implemented. Further research is needed into management and leadership in ambulance care, alongside the ethical challenges in frontline decision‐making under extreme conditions.

**Reporting Method:**

Findings are reported per consolidated criteria for reporting qualitative research (COREQ).

**Patient or Public Contribution:**

No Patient or Public Contribution.

**What does this paper contribute to the wider global clinical community?**

- This study deepens the understanding of the potential for resilient performance through frontline decision‐making, adaptation and learning during an evolving pandemic, thereby contributing to preparedness for future disturbances.
- Under extreme conditions and extraordinary events, such as during a pandemic, dynamic leadership is essential, combining autonomous frontline decision‐making with support from management and clear guidelines.
- Fostering situational awareness, reflection on actions, and engaging in active ethical discourse may strengthen ethical competence, thereby contributing to enhanced confidence in decision‐making during ethically challenging situations.

## 1 INTRODUCTION

The sudden massive outbreak of coronavirus disease 2019 (COVID‐19) led to a global crisis. Healthcare systems worldwide were overwhelmed by the challenge of caring for COVID‐19 patients. During this time, ambulance services, operating on the front lines, were significantly affected by increased demand and the stress of working in a new situation. Due to a lack of resources, difficult prioritizations needed to be made, sometimes conflicting with ethical values (Yuk‐Chiu, 2021). To adapt to insufficient capacity, new protocols and procedures were implemented. Altogether, the extreme conditions led to new experiences, providing lessons to be learned (Brown et al., 2023).

## 2 BACKGROUND

### 2.1 Decision‐making in the complex and dynamic environment of ambulance care

The primary tasks of ambulance care have historically been summarized through four Ts; *Time*, *Triage*, *Treatment* and *Transport*, implying a short response time to the injury site, decision‐making and prioritization of patients by triage (Kennedy et al., 1996), proper treatment, and prompt and safe transportation to a hospital (Porter et al., 2014). Ambulance care requires decision‐making in an unpredictable setting, where advanced care is provided with limited access to higher medical support. The setting allows ambulance teams to meet patients in the familiar environment of their own homes, which may facilitate shared decision‐making and promote attention to the individual patient (Holmberg & Fagerberg, 2010). Compassionate and ethical decision‐making in ambulance care can contribute to creating a caring relationship between an ambulance nurse and a patient, promoting patient participation (Torabi et al., 2018). However, previous studies show that it may be hard to prioritize a caring relationship in the acute stage (Svensson et al., 2019).

With the advancement of care in the out‐of‐hospital setting and an increasing number of non‐conveyed patients (Lederman et al., 2020), the complexity of ambulance care is rising. The role of ambulance nurses has evolved significantly, particularly in a Swedish context. Today, these professionals are not only tasked with on‐scene patient assessment but also with navigating intricate care pathways, reflecting a shift towards more advanced decision‐making and patient care strategies (O'Hara et al., 2015). This complexity was amplified further by the COVID‐19 pandemic (Satty et al., 2021), underscoring the importance of adaptable, informed decision‐making in prehospital settings.

In Sweden, ambulance nurses are highly trained professionals, often with advanced degrees in nursing and specialized training in prehospital care. They operate under local or regional guidelines and protocols, utilizing decision support tools and algorithms to guide clinical decisions, prioritize care, and manage resources (Andersson et al., 2019). This blend of analytical and intuitive decision‐making is vital in dynamic environments where speed is of the essence (Bujold et al., 2022; Forsgärde et al., 2021; Kuziemsky, 2016), emphasizing the unique role that ambulance nurses play in the healthcare continuum (Wihlborg, 2018).

Ambulance nurses rely on a combination of existing knowledge and experience, with the aid of decision support systems (Andersson et al., 2020; Fager et al., 2024). Clinical reasoning and decision‐making are crucial in prehospital settings, due to the substantial risks to patient safety associated with incorrect decisions (Magnusson et al., 2022) and the potential ethical dilemmas they may create (Holmberg et al., 2023). The decision‐making process comes with difficult considerations, trade‐offs and potential risks of bias (Johansson et al., 2022; Yeung et al., 2019). Studies also show that, for ambulance nurses, the process of deciding on non‐conveyance and referring patients to alternative levels of care may be associated with uncertainty and fear of misjudgement (Höglund et al., 2019). High‐stress conditions—typical of emergency care—increase the risks of diagnostic errors and negative patient outcomes (Gandhi & Singh., 2020). However, such conditions also contribute to experiential learning, which can lead to improvements in practice (Dahlin et al., 2018). Thus, studying frontline decision‐making under extreme conditions could deepen the understanding of resilient performance in ambulance care.

### 2.2 Ambulance care and resilience during COVID‐19

The COVID‐19 pandemic can be seen as a massive test for healthcare systems' abilities and weaknesses, with even the most well‐prepared healthcare systems facing insufficient treatment capacities (Rieckert et al., 2021). Resilience engineering represents an evolution in safety and risk management. It defines ‘success’ as the ability of organizations, groups, and individuals to anticipate and proactively mitigate risks before failures occur (Hollnagel et al., 2006). In healthcare, organizational resilience is the ability to maintain stable operations and high‐quality care even under changing and unpredictable conditions. This entails adapting effectively at various system levels in response to evolving challenges (Wiig et al., 2020). An adaptive system is one that not only adjusts and evolves in response to changes but also harnesses experiences to drive continual improvement and innovation. This process of ongoing learning is essential for refining and enhancing system performance over time. However, adaptations may also yield unintended side effects, including resource strain, decreased stability, and possible compromises in quality (Wiig & Fahlbruch, 2019). At the core of organizational resilience is the concept of potential for resilient performance, defined as having the capacity to act in specific ways under certain conditions (Hollnagel, 2018). It involves monitoring and responding to issues efficiently, learning from past events to adapt to new conditions, and anticipating future challenges and opportunities to maintain operational continuity. In the realm of ambulance care, adaptability and resilient performance are paramount, hinging on the ability to anticipate potential risks and establish robust communication among team members. Additionally, consistent adaptation is vital within the inherent dynamic and high‐stress environment of emergency settings. Central to adaptation is the aspect of learning. As situations evolve and new challenges present themselves, a wealth of knowledge and understanding is acquired from decision‐making and adaptations. This process not only enhances skills but also sharpens responses for future scenarios.

## 3 THE STUDY

Working under extreme conditions may increase the risk of suboptimal decision‐making and negative outcomes. The prolonged strain of the COVID‐19 pandemic presented a unique opportunity to study how healthcare systems operate under significant pressure, as regards decision‐making, flexibility, and adaptability. Studying frontline decision‐making and adaptation under extreme conditions may contribute to learning and deepened understanding of resilient performance. To improve preparedness for future healthcare disruptions and crises, this study aimed to explore frontline decision‐making, adaptation, and learning in ambulance care during the evolving COVID‐19 pandemic.

## 4 METHODS

### 4.1 Design

**FIGURE 1:**
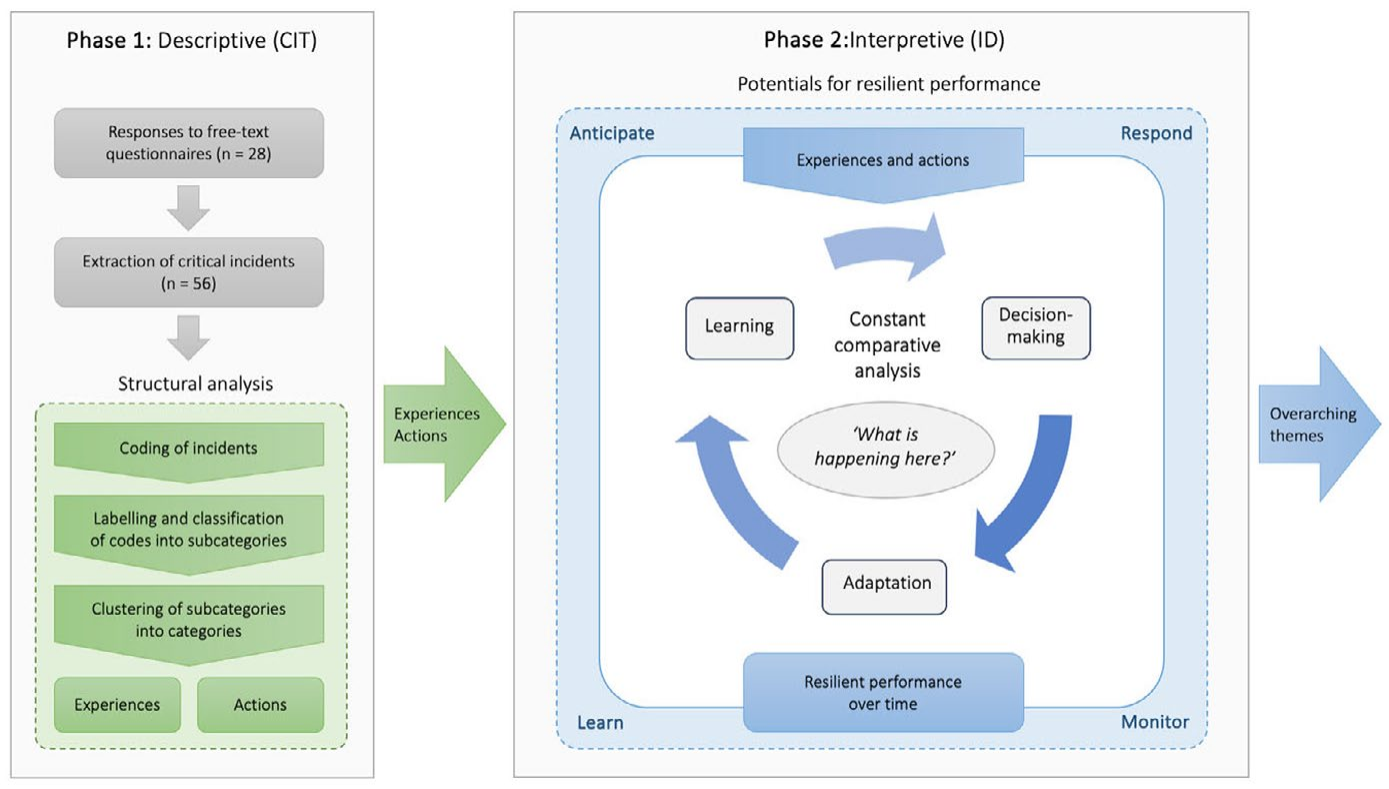
Schematic illustration of the analysis process. Structural analysis using the critical incident technique (Flanagan, 1954). Resilience as a theoretical frame represented by the four potentials for resilient performance (Hollnagel, 2018). Constant comparative analysis in interpretive description (Thorne et al., 1997).

The study employed a descriptive and interpretative qualitative design with a bifold approach, where the critical incident technique (CIT) (Flanagan, 1954) methodology was used together with constant comparative analysis in interpretive description (ID) (Thorne et al., 1997) methodology. The fusion of methodologies in a two‐phase design (Figure 1) provided a deeper analysis with potential for a comprehensive understanding of frontline decision‐making, adaptation, and learning in ambulance care during the evolving COVID‐19 pandemic.

CIT was chosen for its strength in yielding rich narratives through reflections on experiences and actions in situations—positive or negative—influenced by both cognitive and emotional factors (Fridlund et al., 2017). In CIT, the word ‘critical’ does not mean that an incident per se concerns critically ill or injured patients. Rather, a critical incident refers to an event that generates an action that can be determined as critical, or significant, only in retrospect (Fridlund et al., 2017). As ambulance care is made up of clearly defined care encounters, usually with one patient at a time, the CIT is a suitable method to gather individuals' experiences thereof. However, CIT has a recognized limitation in its analysis as it tends to focus on the identification and categorization of incidents (Viergever, 2019) without exploring deeply into the interpretation of these experiences. To strengthen the analysis and further enrich this exploration, ID (Flanagan, 1954) was incorporated. Using ID, the analysis delved into the decision‐making, adaptation, and learning of registered nurses (RNs) against the backdrop of the pandemic, framed by the theoretical lens of resilient performance (Hollnagel, 2018). This interpretive approach guided our analysis within an epistemological orientation that valued the intricacy of human experiences (Thorne et al., 1997). This design intended to illuminate the intricacies of RNs' responses to the pandemic, contributing to an understanding of resilience in ambulance care, informing clinical practice advancements (Jeppesen & Wiig, 2020).

The study is part of a larger project with the overall aim to improve the understanding of how healthcare adapted during the COVID‐19 pandemic (Bjurling‐Sjöberg et al., 2021).

### 4.2 Study setting

This study was carried out in two regions in Sweden, one covering an area of about 11,000 km^2^ with roughly 300,000 inhabitants (density: around 27 inhabitants/km^2^) and the other covering an area of approximately 28,000 km^2^ with a similar population size (density: around 11 inhabitants/km^2^) (Statistics Sweden, 2021). Ambulance services in the regions includes eight respective 11 ambulance stations in rural and urban areas, handling approximately 37,000 and 36,700 assignments per year, respectively (Nysam. Nyckeltal, 2019). In Sweden, the ambulance service organization is governed by the Health and Social Services Act (SFS, 2017) in conjunction with directives from the Swedish National Board of Health and Welfare (SOSFS, 2009) wherein each of the 21 regions is accountable for delivering ambulance care to their inhabitants. As in many other high‐income countries, ambulance care in Sweden has undergone major changes in recent decades and developed to provide qualified emergency care in the prehospital environment with advanced medical treatment methods (Lindström et al., 2015; Wihlborg, 2018). In Sweden, ambulance care is defined as care performed by at least two healthcare professionals in or near an ambulance, where at least one professional is a RN, with or without specialist training (SOSFS, 2009; Wihlborg, 2018).

### 4.3 Recruitment and participants

Our purposive sampling sought to capture a broad spectrum of experiences (Flanagan, 1954) among RNs in ambulance services during the COVID‐19 pandemic. The inclusion criteria were intentionally broad, requiring only active duty in ambulance services, allowing for the participation of RNs from diverse career stages and with differing experiences. The aim was to promote the richness and variety of data, which are essential for CIT and ID analysis (Flanagan, 1954; Thorne et al., 1997).

Regional operation managers at the two regions, overseeing a total of 19 ambulance stations, were contacted by the project management, and informed about the study. After obtaining permission from the managers, the researchers sent an email with a description of the study and a link to the questionnaire to the RNs, to inform them about the study and ask for their participation. The email contained an introductory letter describing the purpose of the study and giving detailed information that clearly stated that participation in the study was voluntary. Submitting the questionnaire was considered to mean consenting to participate in the study. All participants were offered the option of being interviewed instead of responding via the web‐based questionnaire, if they preferred. No participant chose this alternative.

Among the 28 RNs who participated, there was a representative spread across age groups, educational levels, and years of experience, as detailed in Table 1. To provide further context, these demographics were carefully compared against the average data in Swedish ambulance services (Nysam. Nyckeltal, 2019).

**TABLE 1:** Demographics of participants (*n* = 28).

	*n* (%)
Gender	
Female	19 (68)
Male	8 (29)
Other	1 (3)
Profession and education	
RN without specialist education^a^	3 (11)
RN with one specialist education	20 (71)
RN with two or more specialist educations	5 (18)

	Years
Age	
Range	29–66
Median	43
Work experience	
Range	2.5–42
Median	13.5

Abbreviation: RN, Registered nurse.

^a^ Specialist educations included ambulance, anaesthesiology, emergency care, intensive care, or similar.

### 4.4 Data collection

A study‐specific web‐based questionnaire in Swedish was developed by the authors, where each participant was able to share up to five critical incidents. In the questionnaire, each critical incident was explored through 13 carefully crafted questions, drawing upon the framework suggested by Fridlund et al. (2017). The questions were designed to elicit comprehensive narratives of each incident, ensuring a thorough depiction (Table 2). Importantly, one specific question within this set aimed to ascertain the timing of the incident, allowing us to map each response to a particular wave of the pandemic, thereby introducing a temporal dimension to our data. The waves illustrate widely recognized peaks of the pandemic, as informed by Swedish epistemological data on causalities during the pandemic (National Board of Health and Welfare, 2023).

**TABLE 2:** Questions in the web‐based free‐text questionnaire.

When did the incident take place? Choose between the following: during the first wave (March 2020–September 2020), second wave (October 2020–February 2021), third wave (March 2021–June 2021), or after the third wave of the pandemic (after July 2021).
What was your role during the incident?
Where did the critical incident take place?
Describe the critical incident in as much detail as you can.
What did you do in connection with the incident?
What were your thoughts during and after the incident?
What were your feelings during and after the incident?
What did you find was the most demanding aspect of the incident?
How did this incident deviate from normal work?
Why was this incident critical for you?
What has the incident meant to you since it occurred?
What were the consequences for the patient?
What has the incident meant for your work or the organization since it occurred?

A pilot questionnaire (not included in the results) was tested on two ambulance nurses prior to data collection. Feedback from testing was used to clarify the questions. Data were collected from November 2021 to August 2022, yielding a total of 56 reported incidents (mean = 2 incidents/participant). The collected data encompassed 77 pages of text, providing a rich and detailed narrative of the critical incidents experienced by the participants. During the data collection, two reminders were sent out by email. After reviewing approximately 50 incidents, recurring patterns began to emerge in the data, suggesting that additional incidents were unlikely to provide new critical information (Flanagan, 1954). Consequently, no further reminders were sent out beyond this point.

### 4.5 Data analysis

The analysis unfolded in two structured phases, each designed to leverage the strengths of CIT and ID while addressing their respective limitations.

#### 4.5.1 Phase 1: Descriptive structural analysis using CIT

In the first phase, a descriptive structural analysis was conducted, guided by CIT (Flanagan, 1954). The analysis involved anonymizing the questionnaire responses and conducting a detailed review of the narratives to immerse ourselves in the data. This process revealed the richness and contextual depth of the incidents described, offering insights into the evolving challenges faced by ambulance nurses during different stages of the pandemic. Next, each incident was identified with a clearly delineated beginning and end, where the intention behind the actions taken as well as their impact were defined based on a clear positive or negative outcome. The incidents underwent a structural analysis in accordance with Flanagan (Flanagan, 1954). Excerpts were coded and then classified into subcategories (example in Table 3). These subcategories were given labels and then clustered into categories separated by differences and similarities. Next, a comprehensive structuring of the categories was performed, to separate subjective descriptions of critical incidents into experiences and actions triggered to handle these incidents.

**TABLE 3:** Examples of experiences and actions identified in the CIT analysis process.

Quotation	Subcategory	Category
Experience:		
‘Under the stress cone, my glasses fog, and sweat dampens my shirt beneath the protective gear. A persistent headache looms. We navigate through the night with flashing blue lights, arriving at a tightly packed, temporary COVID‐ICU using outdated ventilators. I get an apocalyptic feeling and I think about how it will end and if we'll be able to work it out.’	Working under pressure with no end in sight	Enduring persistent strain
Action:		
‘During the pandemic, I really struggled with the choice to not transport patients. If there was another safe way to get them where they needed to go, I tried to do that instead. Some people said we should only do our specific jobs and if we run out of ambulances, it's someone else's problem. But I knew all the other ambulances were busy, and I felt I had to think about what that meant for being ready to help others. This gave me a lot of stress because the way I chose to act could affect more than just me or the patient. I felt pressed between different competing values.’	Upholding capacity and readiness in organization	Prioritizing through trade‐offs between caring and efficiency

#### 4.5.2 Phase 2: Interpretive description

In the second phase, the discerned experiences and actions were interpreted in accordance with ID (Thorne et al., 1997, 2004), through the lens of ‘potentials for resilient performance’ (Hollnagel, 2018), which included responding, monitoring, learning, and anticipating events along the pandemic's timeline. This approach aligned with ID's emphasis on iterative and constant comparative analysis (Thompson Burdine et al., 2021), asking the central question ‘What is happening here?’ (Thorne et al., 1997) to uncover underlying patterns and meanings (Figure 1). The constant comparative analysis involved six methodological cyclic steps: immersion in the data, development of an initial thematic template, organization of the data based on the template, condensing of data, and reflecting, and comparing and contrasting data (Thompson Burdine et al., 2021). Throughout these cycles, the questionnaires were revisited to capture the richness of the original data, ensuring the analysis remained deeply rooted in the participants' narratives. This enabled probing deeper into the processes of decision‐making, adaptation, and learning exhibited by RNs during the evolving COVID‐19 pandemic. The cycles of analysis, discussion, and refinement distilled the data into four overarching themes.

This second phase of the analysis also involved relating experiences and actions along the timeline of the pandemic, illustrated by the first, second and third wave. This linkage was facilitated by responses to the question on timing within the questionnaire. This temporal anchoring allowed the tracing of the dynamic nature of the crisis and its impact on emergency care, enriching the understanding of how the ambulance service's responses were adapted over time.

The interpretation process was guided by both the theoretical framework of the study and a grounded approach to the data, ensuring that the themes were not only theoretically sound but also deeply rooted in the empirical data. In line with interpretivism, themes were not considered to be hiding in the data waiting to be discovered. Rather, themes surfaced during the process of the researchers' engagement with the data in the attempt to address the research question. The combination of CIT and ID enabled the capture of both the structural and interpretive dimensions of the participants' experiences, contributing valuable insights into resilience and adaptive practices in the challenges of the evolving COVID‐19 pandemic.

### 4.6 Ethical considerations

The study was in line with the Helsinki Declaration and approved by the Swedish Ethical Review Authority (Ref. No. 2020‐04187). Data gathered in the study were handled in accordance with the EU General Data Protection Regulation (GDPR). No sensitive personal data were collected.

### 4.7 Rigour and reflexivity

The trustworthiness and transferability of the findings were promoted through researchers' reflexibility and rigour in analysis, where relevant principles for sample selection, data collection, and analysis were observed (Thorne et al., 1997, 2004). In interpretive research methodology like ID, the researcher plays a vital role as an instrument within the research (Fridlund et al., 2017; Thompson Burdine et al., 2021). A researcher gains access to understanding the beliefs, motivations, and reasoning of individuals within their social context through social constructions such as language, consciousness, personal experiences, and shared meanings. These elements serve as major sources of insight, shaping the process of interpreting and understanding the data (Thorne et al., 2004). The author group comprised both researchers with extensive experience from ambulance care and researchers without any ambulance experience. The previous knowledge and professional experiences in the author group were critically integrated with the emerging findings, ensuring that personal insights enhanced, rather than predetermined, the research outcomes (Thompson Burdine et al., 2021). Dependability was considered by returning to the data with themes, categories, subcategories, and codes, and performing constant comparisons of emerging concepts. To promote credibility, the result of the analysis was discussed, challenged, and redefined by all authors. Member checks and independent scrutiny were incorporated into the research process (Thompson Burdine et al., 2021), as the researchers shared emerging findings with peers, active RNs, and other healthcare experts in another region to challenge and confirm evolving interpretations (Thorne et al., 2004). To further strengthen confirmability and credibility, the results were supported with quotes taken from the participants' responses, selected based on the identified themes, the intensity of the described experiences, and their ability to communicate the narratives. Linguistic accuracy was promoted through a dual‐check translation process. This involved initial translations of responses in Swedish into English by professional translators, followed by back‐translations from English into Swedish by bilingual healthcare professionals on our team. This approach not only ensured the precision of context and terminology, but also preserved the cultural nuances and integrity of participants' narratives.

## 5 FINDINGS

The frontline decision‐making, adaptation, and learning in ambulance care during COVID‐19 pandemic was interpreted into the themes ‘Navigating uncharted waters under never‐ending pressure’, ‘Balancing on the brink of an abyss’, ‘Sacrificing the few to save the many’ and ‘Bracing for the next wave’. The themes are presented together with the corresponding categories and subcategories from the structural analysis (Table 4).

**TABLE 4:** The findings presented as subcategories, categories, and themes.

Subcategories	Categories	Themes
Fearing for life of self and family Realizing own mortality Taking pride in saving lives Acknowledging others' efforts	Fearing for life while saving lives together	Navigating uncharted waters under never‐ending pressure
Uncertainty and lack of knowledge Overwhelming information flow Lack of support and governance Limited resources and capacity Redeployment of competence	Working in challenging and uncertain conditions	
Temporary respite in endless efforts Emerging fatigue and manifest fatigue Working under pressure with no end in sight Getting accustomed to a new normal	Enduring persistent strain	
Caring or uncaring patient encounters Difficulty providing high‐quality care Dehumanizing interactions Difficulties in coping	Performing at the boundaries of care	Balancing on the brink of an abyss
Responding to ethical demands while ignoring own needs Stepping back from care to shelter oneself	Protecting or sacrificing oneself	
Upholding capacity and readiness in the organization Introducing unorthodox solutions and workarounds Acting as a gatekeeper to advanced care Embodying ethical responsibility in decision‐making	Prioritizing through trade‐offs between caring and efficiency	Sacrificing the few to save the many
Substituting technical skills with ethical values Being there as a human being when all else fails	Re‐defining the concept of best possible care	
Detecting high‐risk situations and threats to patient safety Overriding the safety margin Identifying and abandoning maladaptations Returning to conventional work methods	Learning from successes and failures	Bracing for the next wave
Adopting new guidelines Performing within limited margins Adapting technical equipment Introducing new equipment after rapid training Preparing for a new wave	Anticipating and adapting to continuous change	

**FIGURE 2:**
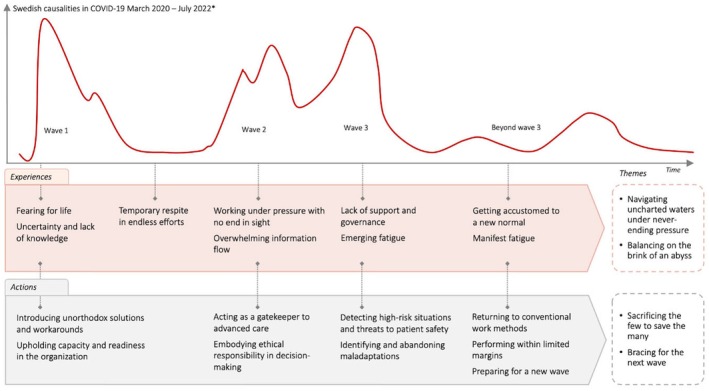
Resilient performance by the process of decision‐making, adaptation, and learning during the evolving COVID‐19 pandemic. * National Board of Health and Welfare (National Board of Health and Welfare, 2023).

Drawing from the experiences, actions and overarching themes, the findings illustrate resilient performance linked to the processes of decision‐making, adaptation, and learning throughout the waves of the COVID‐19 pandemic, as illustrated by the timeline in Figure 2.

### 5.1 Navigating uncharted waters under never‐ending pressure

This theme highlights the decision‐making and adaptation challenges that RNs encountered while striving to save lives alongside fellow professionals, all the while being concerned for their own safety. Providing ambulance care during the pandemic was described as working under persistent strain in a situation ‘more terrifying than anything else’. Faced with demanding and unpredictable conditions without previous knowledge to relate to, RNs grappled with navigating uncharted waters.

RNs faced overwhelming scenarios that challenged their decision‐making, adaptability, and ability to learn. Phrases like ‘living in a nightmare’ (participant 5) embodied their fears, not just for patients, but also for their own safety and that of their loved ones. This palpable dread sometimes clouded their decision‐making processes, knowing each decision might lead to unanticipated consequences. Amidst these challenges, they had to balance personal fears with their professional responsibilities. Confronting younger patients who were critically ill or died from the virus made the RNs feel powerless and realize their own mortality:

Responding to a call about a patient on day nine of illness, I was confronted with a man in his 30s—previously healthy. Now, struck down by COVID, he was noticeably affected, showing low oxygen saturation and a terrified gaze. It was my first encounter with a severely ill young patient. I remember the fear in his eyes and the profound sense of powerlessness that washed over me. That moment imprinted on me the fear and the stark reality of what COVID could do to a person. (Participant 1)

Such encounters necessitated a rapid adaptation in RNs approach to care. They had to balance their natural desire to help with the tough realities of the COVID‐19 pandemic. Their ability to adapt was directly linked to continuous learning—from understanding the novel nature of the virus, adapting to changing protocols, to navigating the emotional and psychological challenges it presented. Split‐second decisions needed to be made, often in the face of overwhelming uncertainty and emotional distress. Yet, these challenges became a possibility for both personal and professional growth. Over time, these experiences shaped and refined their clinical judgement, resilience, and coping strategies, even if the sense of powerlessness persisted.

The pandemic forced RNs to make crucial decisions without the guidance of past experiences or established protocols. Their usual frameworks and resources became obsolete amidst the ever‐changing challenges of COVID‐19. The feeling of being unprepared was amplified by a lack of robust decision‐support systems. The phrase ‘many questions, few answers’ (participant 10), encapsulated their dilemma:

What treatment guidelines do we have? We had none. What can I do for these patients? She got oxygen, but could more be done? Does inhalation help? So many unanswered questions. We didn't have guidelines initially; they came later. (Participant 28)

After an initial lack of guidelines, new information was distributed in an overwhelming manner, and it was considered hard to keep track of the most recent rules and regulations. The RNs experienced work during the COVID‐19 pandemic as navigating uncharted waters under never‐ending pressure, with conditions and guidelines changing rapidly from day to day. The decision‐making was experienced as requiring continuous adjustments, without clear instructions or a mandate from management. Due to the lack of clear leadership, the RNs felt abandoned by management in this extraordinary situation: ‘We really need support and governance from management, but where are they? They are invisible’ (participant 20). Furthermore, the shortage of resources and capacity in the healthcare system during the pandemic was perceived to affect the ambulance service organization greatly and was described as an experience of incapability. Limited transport capacity called for redeployment solutions, where RNs with additional specialist education in intensive care or anaesthetic care needed to take on unfamiliar roles during transfers of patients with only a brief introduction, putting added stress on the individual.

After the first wave, there was a temporary respite, with a decline in the spread of COVID‐19 during the summer of 2020. This was described as a momentary relief before contagion started all over again. After this respite, it was difficult to muster energy and endurance, as fatigue was starting to emerge. Having worked under pressure with no end in sight for an extended period, the RNs described feeling demoralized. After enduring persistent strain, both physically and mentally, the question ‘when will it ever end?’ (participant 1) arose, conveying a sense of hopelessness. However, at the end of the pandemic, the RNs had adapted to the situation as a ‘new normal’ and stated that ‘this is the way we work now’ (participant 28).

Despite the hardships, there was also a sense of purpose and accomplishment. The RNs felt honoured to serve on the front lines during such a historic event, working together with other professions, saving lives. They expressed gratitude and respect for others in the healthcare field, especially those in social care roles who provided care in homes, often with insufficient personal protective equipment (PPE).

### 5.2 Balancing on the brink of an abyss

In this theme, RNs found themselves on the edge, constantly assessing the fine line between delivering care and self‐preservation. They were metaphorically balancing on the brink of an abyss, where a single misstep—or failed decision—would lead to consequences for both them and their patients. The theme illuminates the ethical considerations and challenges that healthcare professionals may face in their efforts to provide optimal care while grappling with personal, moral, and systemic challenges. It captures the importance of compassion and humanity in patient encounters, the ethical dilemmas involved in performing at the boundaries of care, and the potential failures in delivering high‐quality care under extreme conditions.

The work in ambulance care during COVID‐19 was a balancing act, pushing the boundary on acceptable work performance. Patient encounters were experienced as caring or uncaring, depending on the possibility to provide medical treatment and the level of human interaction achieved in the process. The work conditions were experienced as contributing to difficulties in providing high‐quality care, which led to ethical stress. A genuine commitment to each patient as a person was hard to muster as the RNs were running on reserves, at times resulting in dehumanizing interactions. The lack of energy and motivation left no other option than adapting to the situation and doing the bare minimum at work. While being used to physical work and heavy lifting, the RNs experienced an additional dimension to this, as they were often freezing or sweating because of the PPE and hygiene restrictions. Wearing extensive PPE led to difficulties in coping, making it hard to genuinely engage with patients. The PPE was also perceived as creating a barrier to communication and contributing to impersonal encounters:

You viewed all the patients as if they were lepers. You greeted them from a distance […] That meant that you never got that empathy aspect, really. It's such an important part of care in other cases, just simply meeting as two individuals […] that building of a sense of trust, the human part of the meeting and the physical contact. (Participant 17)

Work was also experienced as a balancing act between protecting and sacrificing oneself to respond to the ethical demands in patient encounters. As the RNs faced patients in critical need of help, they were confronted with decision‐making in responding to the ethical demands without having time to put on PPE, ignoring their own needs. Stepping back from care to protect oneself first would create a stressful situation, with the patient having to wait for help.

In the incidents described by the RNs, there were also signs of how compassion and humanity reached beyond the physical barriers of PPE, contributing to caring encounters. For patients who had been alone in their homes for months, human touch could mean everything:

He hadn't had any human contact or physical touch in the last few months and completely broke down when I, with all my protective equipment, face shield, gloves, and mask, took his hand. He didn't want to let go. (Participant 27)

### 5.3 Sacrificing the few to save the many

This theme encapsulates how actions and adaptations in ambulance care during the pandemic were responses to the imbalance between demands and capacity. Prioritizations were made by the RNs in a trade‐off between caring and efficiency, to uphold capacity and readiness in the organization. Feeling responsibility for ensuring ambulance availability and resources to provide care for local citizens meant that decision‐making was influenced by the need to be available for other assignments. Hence, ordinary guidelines were sidestepped during the pandemic as unorthodox solutions, workarounds, and quick fixes were introduced. This adaptation could solve problems and reduce the time needed for setting up the ambulance and technical equipment but was often based on stopgap solutions. To address competing priorities and save as many lives as possible, the RNs would sometimes need to prioritize higher‐level goals, such as maintaining capacity for the many, over lower‐level goals, like their own wellbeing or the patient in front of them.

Without support and governance from management, the RNs pressured themselves to make trade‐offs between caring for each patient as a person and providing efficient care to a larger population. Providing efficient care with limited resources thus meant needing to sacrifice the few to save the many. The RNs were engaged in decision‐making to determine which patients were to receive what care. By prioritizing patients to the appropriate level of care, the RNs could promote the greatest gain for the many. Adhering to guidelines at the emergency department meant refusing next of kin the opportunity to accompany a patient in the ambulance, which was often allowed before the pandemic. When a patient was anticipated to be in such a bad state that death could be imminent, preventing a last goodbye or the presence of a family member by the patient's side created ethical stress in the RNs. Refusing to accept patients into care meant sealing their fate, underscoring the ethical responsibility inherent in decision‐making. Such processes could induce ethical stress through the realization of how a decision could affect not only one patient, but also others waiting for care. Older patients with COVID‐19 were seldom welcome at the hospitals or at short‐term care facilities. ‘So, where should the patient go? They are not welcome anywhere’ (participant 17). At the same time, as older patients were transported from nursing homes to hospital, the RNs grappled with the concern that their actions might contribute to patients ending up in non‐caring environments with limited human contact, which raised questions regarding the appropriateness of their decision‐making:

I have been trying to support the physicians in their tough decisions regarding which patients should be given a chance, but sometimes it has been challenging to stand behind their choices … It's completely insane that patients must be prioritized in terms of the level of care they should have access to. He didn't get a fair chance. He was brought in simply to die in chaos and solitude. (Participant 13)

Thus, RNs served as ‘gatekeepers’, determining which patients were eligible to go to advanced care, which meant denying others. Lacking the usual alternatives, as the medical treatment available was insufficient, the RNs acted by reaching out as fellow human beings, substituting technical skills with ethical values. They could hold a patient's hands to comfort them, be there for them as a human being when all else failed, and re‐define the concept of best possible care:

I felt powerless … His eyes were terrified throughout the 30‐minute‐long transportation. The only thing I could do was give him oxygen and hold his hand. (Participant 1)

### 5.4 Bracing for the next wave

This theme highlights the potential for learning from both successes and failures when adapting to extreme conditions. It captures how working under continuous change brings a need to constantly adapt and brace for the next disturbance and shows the importance of identifying high‐risk situations created by adaptations. Further, it illuminates how adaptations may result in threats to patient safety and how abandoning maladaptations is an important step in resilient performance and delivery of safe care.

During the pandemic, the RNs at times detected high‐risk scenarios with threats to patient safety which had inadvertently arisen due to their prior actions and adaptations. Thus, these actions had led to unintended consequences and overrode the safety margins, rather than yielding the desired outcomes. During the first wave of the pandemic, the RNs were working blindly without clear guidelines, trying to handle the situation, and testing different working methods. This response was reactive, based on the scarce knowledge and routines available to handle the new threat. Initially, this meant using a trial‐and‐error approach, changing from day to day, fostering learning as to what worked and what did not.

During the second wave, the RNs were equipped with lessons learned from the initial wave; they had honed their skills and gained heightened proficiency in the work. The patient group with COVID‐19 was not unknown anymore; the RNs knew what to do and a more proactive approach evolved. Both successful and unsuccessful adaptations were implemented. One adaption that became adopted as a new guideline was the use of oxygen saturation monitors for a so‐called ‘walking‐test’, which aided the RNs in the decision on whether a patient needed in‐hospital care. As resources were still lacking, trade‐offs were made to increase efficiency and uphold capacity. For example, adaptations included a single RN to be used in patient encounters, instead of the normal dyad. Furthermore, so‐called ‘stripped’ ambulances with only the bare minimum of equipment were introduced for transportation of COVID patients, to save time and increase efficiency. However, the use of solo work and stripped ambulances was later found to breach the safety margins and create risks for both RNs' own safety and that of patients, as the RNs found themselves lacking important support and equipment:

We stopped using a stripped ambulance after a while, because we didn't know until we reached the patient what type of equipment we needed. Instead, we used our regular ambulance and both nurses in the ambulance team went to the patient and examined them, in accordance with our treatment guidelines. (Participant 9)

During the third wave, learning from previous maladaptations such as solo work led to improvement of patient safety. The RNs returned to conventional work methods, such as two‐person work and fully equipped ambulances. Guidelines were implemented that aided in distinguishing COVID‐19 from other conditions.

After the third wave, work returned to normal, but with limited resources. Efficient work methods with fast‐track decontamination procedures were developed, but COVID‐related assignments still took longer, testing the margins of the organization's available resources. With limited margins in the organization, the RNs worked overtime, covering for ill colleagues to maintain capacity in the ambulance service, despite needing recovery themselves. The RNs gained crucial insights of the contagion over time. Awareness and routines in the use of PPE emerged as a lesson learned, contributing to better hygiene routine adherence. Improved routines at the emergency dispatch centre promoted preparedness for the RNs, whether a patient exhibited infectious symptoms. The RNs anticipated potential disturbances and the need of additional resources in both the short‐ and the long‐term by adapting to the continuous changes. Technical equipment was adapted, and new or alternative equipment was acquired and implemented to circumvent the shortage of material resources, with rapid training provided so the equipment could be put into use immediately, in preparation for the possibility of a new wave. Furthermore, the dissemination of information from management to the frontline workers was improved, as new methods for digital information exchange were implemented, showing examples of organizational learning from the challenges of the pandemic.

## 6 DISCUSSION

Exploring frontline decision‐making, adaptation, and learning in ambulance care under the extreme conditions caused by the evolving COVID‐19 pandemic has shed light on the myriad obstacles faced by RNs. Confronted with the unprecedented threat of a pandemic, the RNs feared for their own safety in what felt like navigating unchartered waters under never‐ending pressure. Unable to provide high‐quality care to the degree to which they were accustomed, the RNs were caught in ethical dilemmas in what can be described as balancing on the brink of an abyss. A tidal wave of demand, unleashed by the pandemic and paired with insufficient resources, necessitated heart‐wrenching prioritizations—occasionally sacrificing the needs of a few to save many. The COVID‐19 pandemic presented disruptions in daily work which created challenges to persistence, sustainability, and resilience in the ambulance service organization. The RNs, being aware of the extensive challenges ahead, braced for each new wave of the pandemic, attempting various adaptations to preserve resources and operational capacity, preparing with what resources they had. However, with limited possibilities for recovery, signs of fatigue emerged early in the process.

### 6.1 Navigating conflicting values in pandemic‐driven decision‐making

Responding to the discrepancy between demand and capacity during the COVID‐19 pandemic situated the RNs in vulnerable situations. Decision‐making with available knowledge and resources meant wrestling with conflicting demands and emotional strain. The RNs frequently found themselves weighing the urgent needs of individual patients against the overarching capacity of the ambulance service organization, a predicament reflective of prior studies (Brown et al., 2023). Decision‐making during this period often embraced a utilitarian moral stance, sometimes necessitating ethical trade‐offs and the sacrifice of the few to secure greater benefit for the many (Beauchamp & Childress, 2013). Individual needs occasionally took a backseat to uphold societal responsibility, with a discernible impact on older patients. RNs were confronted with the ethical obligation to recognize and respond to others' needs as argued by Lögstrup et al. (2020), which, in practice, translated into the tangible moral difficulty of meeting patients, recognizing their needs, but being potentially unable to assist. The ethical dimensions within ambulance care extend beyond the immediate confines of the prehospital care environment, unfolding expansively with every unique human interaction and permeating beyond the immediate clinical contexts (Holmberg & Fagerberg, 2010). Consequently, fostering ethical competence within the organization demands the establishment of a structured framework for ethical reflection, which, in turn, fortifies professional identities and cultivates shared ethical values (Holmberg & Fagerberg, 2010).

The RNs experienced physical and emotional strain, often prioritizing patients' needs, even at a cost to their own well‐being and that of their families and colleagues, aligning with findings from other studies (Morley et al., 2020). Performing uncaring encounters could create subsequent conscience stress for RNs, especially amidst the awareness of not delivering high‐quality care while dealing with their own exhaustion. Compassion fatigue—an erosion of empathy due to dealing with high‐intensity emotional workload without adequate time to recuperate (Decety, 2020), contributing to an inability to nurse others (Nolte et al., 2017)—began to manifest, culminating, in some instances, into discernible burnout. In the pandemic's latter phases, the RNs narratives recurrently described a demoralizing, seemingly unending struggle, underscoring a burnout risk. Amidst visible signs of emerging fatigue, the lack of opportunities for recovery and restitution (Ekstedt & Fagerberg, 2005) meant RNs had to persevere, propelling some towards palpable fatigue. This scenario is crucial, given the linkage between exhaustion, intent to leave the healthcare profession, and actual departures from the field, as illuminated in various studies (Abbasi, 2022; Dyrbye et al., 2021; Havaei et al., 2016; Vuilleumier et al., 2023).

Working on the front lines meant RNs often operated as ‘gatekeepers’, implementing physician orders concerning the conveyance of older patients from nursing homes to hospitals, with noticeable increases in non‐conveyances during the pandemic (Satty et al., 2021). Decision‐making might pivot on prioritizing hospital capacity for those with higher survival probabilities or shielding vulnerable demographics from infection. Nevertheless, benevolent intentions like lockdowns and quarantines inadvertently resulted in detrimental collateral damage, particularly among older patients aged 65 and above (Vuilleumier et al., 2023). Being actively involved in determining a patient's fate, with limited ability to do what was perceived as morally right, would cause ethical stress. This situation was exacerbated when RNs, devoid of substantial management support, found themselves singularly navigating these harrowing decisions. More manageable were instances where decision‐making was scaffolded by regulations, routines, or decision‐support tools, as it lifted the burden from individual RNs in determining patient conveyance to hospitals. The institution of guidelines grounded in evidence fosters the potential for safeguarded and efficient prehospital care (Hagiwara et al., 2013).

### 6.2 Navigating safety during the waves of a pandemic

The unprecedented threat of the pandemic created a pervasive atmosphere of fear and uncertainty among the RNs. Adhering to a ‘better safe than sorry’ philosophy, the RNs prioritized self‐protection through all available means until further knowledge was acquired. In retrospect, this behaviour is understood to have hindered the ability to engage in caring encounters and to view each patient as a unique individual, a cornerstone of nursing care (Holmberg & Fagerberg, 2010).

During the waves of the pandemic, the RNs dynamically adjusted to shifting needs and challenges. While they navigated through the large influx of patients by improvising with new, often untested, work methods, the consequent sidestepping of established guidelines becoming routine. However, this adaptive response—driven partly by a compelling focus on identifying COVID‐19 symptoms—simultaneously elevated the risk of overlooking other pertinent medical conditions in patients, confirming outcomes of previous studies (Satty et al., 2021; Vuilleumier et al., 2023).

The findings illustrate that some adaptive measures, while problem‐solving in nature, inadvertently introduce increased risks to both patients and RNs. Successful adaptation, as underlined by resilient performance theory, necessitates more than ad‐hoc adjustments (Lyng et al., 2021). Efforts to uphold operational capacity despite constrained resources led to a shift in what was considered acceptable performance, pushing the organization to move the safety margins, operating at the edge of safety—a phenomenon referred to as ‘flirting with the margin’ (Rasmussen, 1997). This tenuous scenario not only increased the likelihood of failure but also cascaded consequences across all system levels. Under extreme situations, the organization's safety is tested, revealing underlying systemic brittleness. Though certain adaptations aimed at enhancing long‐term efficiency and aiding a broader patient pool, they paradoxically precipitated a short‐term decline in care quality. This aligns with existing research asserting that adaptations, even when locally successful, can induce system brittleness (Lyng et al., 2021).

However, we found that within the confines of a pandemics' extreme conditions, adopting an ‘adaptive trial‐and‐error’ approach could be indispensable for enabling evolution and foster learning from both successes and failures. Engendering a culture of reflexivity within work groups can underpin the assessment process, determining the efficacy of adaptations. An illustrative example being the transition back from solo to dyad work, aimed at safeguarding conditions. This action mirrored a palpable potential for resilient performance, illustrating that amidst challenges, the potential for systemic learning and improvement endures.

### 6.3 Navigating the waves: Frontline resilience and leadership challenges during the COVID‐19 pandemic

Under extreme conditions, at the verge of chaos, prompt decisions need to be made. Thus, in the complex context of ambulance care, dynamic leadership is needed, combining a mandate for autonomous decision‐making at the front lines with support from management and clear guidelines. The dynamic leadership model proposed by Hopkins (Hopkins, 1999), which emphasizes clear mandates and necessitates leaders to directly support frontline workers in decision‐making, serves as a pertinent example. Under extreme conditions, such as during pandemics, leadership should support the frontline healthcare professionals with robust management systems and decision support. To align demand and capacity, the vertical communication in the system becomes pivotal, with reflection having a central role (Svensson et al., 2023).

When the first wave of the COVID‐19 pandemic struck Sweden, the ambulance service found itself unprepared, lacking critical resources such as PPE and clear work guidelines for navigating the pandemic, thereby necessitating a reactive approach. RNs, accustomed to navigating the dynamic environment of ambulance care, typically adapt their responses to immediate situations (O'Hara et al., 2015). While resilient performance encompasses the ability to respond to disruptions rapidly and effectively by assessing the situation and taking the appropriate action, a system is not inherently resilient. Rather, it can hold the potential for resilient performance (Hollnagel, 2018) through its adaptive capabilities—anticipating, adjusting, or modifying operational functions as per extant conditions—which necessitate a blend of responsive and learning abilities, and perhaps monitoring capacities as well.

Nonetheless, the findings indicate that it was not the ambulance service organization, but rather the individual RNs, who embodied the potential for resilient performance, assuming responsibility to preserve operational capacity by stretching their own limits in the absence of substantial governance and managerial support. When the resilient performance in an organization primarily depends on individual adaptability, it may lead to the appearance of a functioning and resilient organization, as the problems are solved. However, this ‘tragedy of adaptability’ may conceal brittleness and safety risks within the system (Wears & Hettinger, 2014). Operating at a breaking point, RNs, already buckling under the strain, expressed concern regarding the organization's ability to manage further disturbances, given its existing fatigued and fragile state, should another crisis unfold.

Amidst the pandemic, where evidence was scant, the role of management in decision‐making became paramount to alleviate moral stress in frontline decision‐making and mitigate the risk of preventable harm. A strong need for clear leadership emerged, yet it was perceived to be notably absent. The RNs sought guidance from management, which found itself grappling with the formidable challenge of making informed top‐down decisions without access to evidence‐based knowledge. Consequently, leaders had to navigate decision‐making while frontline professionals were pushed to their limits. Emerging Swedish investigations indicate a deficiency in evidence‐based practice (Lederman et al., 2018) and a shortage of national guidelines for ambulance care (The National Board of Health and Welfare, 2023). Therefore, under extreme conditions, important areas of improvement are reinforcing proactive management participation and guidance, coupled with the further development of evidence‐based decision support.

Our findings echo global challenges faced by healthcare workers, as seen for example in Australia's system response during COVID‐19 (Clay‐Willams et al., 2020), and the ethical dilemmas and compassion fatigue arising worldwide (Fox & Meisenberg, 2022; Løgstrup et al., 2020). A study in Ireland emphasized the need for frontline support (Brown et al., 2023), whereas research on emergency service demands has highlighted the pandemic's extensive impact (Vuilleumier et al., 2023). These comparisons underline the critical need for resilience and adaptability in emergency healthcare globally.

### 6.4 Strengths and limitations

This study provided insight into frontline decision‐making, adaptation, and learning during an evolving pandemic, utilizing a combination of methodologies to illuminate the multifaceted experiences of participants. However, it is important to acknowledge the inherent limitations associated with retrospective methodologies, such as the CIT, particularly as regards memory recall (Fridlund et al., 2017). Still, the reflective aspect is not simply a limitation—it is integral to the ontological and epistemological foundations of qualitative research, acknowledging that participants' reflections are shaped by their evolving understanding.

In CIT methodology, a target of 50 incidents is suggested as a lower limit (Flanagan, 1954). Recognizing the critical role of ambulance workers as multifaceted, the collection of 56 incidents could be perceived as limited. Although more incidents could potentially have provided a broader view, the quality and specificity of the incidents reported (Schluter et al., 2008; Thompson Burdine et al., 2021; Viergever, 2019) were deemed to provide significant insights into the complexities of the situations faced by RNs during the pandemic.

Although the web‐based questionnaire limited opportunities for follow‐up questions, it enabled widespread participation within the organizations (Schluter et al., 2008). Acknowledging the potential for richer data from interviews, we systematically evaluated the narratives for depth and breadth. This assessment, based on qualitative criteria such as thematic saturation and the diversity of insights, led us to collectively determine that the data density satisfied our research needs. Consensus was reached through a critical review of the collected narratives, ensuring that they provided a detailed and comprehensive understanding of the incidents explored.

A longitudinal design, capturing data throughout the pandemic, might have provided more robust insights. However, our ability to access and recruit participants was limited, particularly within ambulance services, where fatigue and exhaustion were prevalent. This challenge in participant recruitment underscores the study's findings, highlighting the extreme conditions faced by frontline workers and validating our conclusions through the difficulties encountered in the research process itself.

Trustworthiness and transferability of findings were promoted through researchers' reflexibility and rigour in analysis, as CIT was extended through the use of ID methodology, providing the flexibility to transcend the disciplinary boundaries of conventional methodological approaches (Thorne et al., 2004).

The sample composition in our study mirrors the contemporary landscape of Swedish ambulance services (Statistics Sweden, 2021), providing contextually rich data that bolster the validity of our findings. Consequently, the study's results may be transferrable to contexts within Sweden and to neighbouring countries with similar welfare structures. Although the transferability of the results on a broader international scale might be limited due to structural variances among ambulance service organizations worldwide, it is presumed that human experiences and resulting actions under extreme conditions share similarities, transcending specific contexts.

### 6.5 Implications for policy and practice

The knowledge gained from this study deepens the understanding of frontline decision‐making, adaptation, learning during an evolving pandemic, which may be of value in preparing the ambulance services for future disturbances. Important areas of improvement to safeguard patient safety are increasing the active participation and governance from management under extreme conditions and implementing decision support and guidelines to support frontline healthcare professionals in their work.

In conditions that necessitate prioritizations, older patients may suffer collateral damage. During pandemics or other unexpected disasters, decision support and interventions targeting this population should be considered a high priority. Strengthening ethical competence and developing the ethical discourse on prioritizations, for example through simulation training, could promote confidence in decision‐making under ethically challenging or extreme conditions.

The study shows the importance of vertical decision‐making as a dynamic interaction between management and frontline professionals. Further research is needed on the perspectives of management and leadership in ambulance care to support decision‐making, adaptation, and learning under extreme conditions.

## 7 CONCLUSIONS

At the verge of chaos, such as during a pandemic with a novel disease, the necessity for prompt decision‐making under extreme conditions becomes evident. Insufficient resources may require making difficult prioritizations, re‐negotiating the concept of high‐quality care. However, limited possibilities of responding to patients' needs may increase ethical stress and can contribute to fatigue. When the available knowledge base is limited and guidelines are lacking, learning arises as frontline healthcare professionals grapple with incorporating new information and transforming experiences into fresh insights and practices through reflexive adaptations and decisions. This highlights the crucial role of individual initiatives to make difficult decisions in ethically demanding situations, and the importance of taking advantage of lessons learned and knowledge acquired by decisions and adaptations made in response to challenges. Additionally, in the dynamic context of ambulance care, dynamic leadership is needed, including a combination of a mandate for autonomous decision‐making on the front lines, support from management, and clear guidelines.

## AUTHOR CONTRIBUTIONS

All authors have agreed on the final version of the manuscript and meet at least one of the following criteria: (1) substantial contributions to conception and design, acquisition of data or analysis and interpretation of data, and (2) drafting the article or revising it critically for important intellectual content.

## FUNDING INFORMATION

The Kamprad Family Foundation for Entrepreneurship, Research, and Charity (Grant No. 20190249), the Centre of Clinical Research Sörmland, Uppsala University, Sweden (Grant No. DLL‐940876), and the Regional Research Council in Mid Sweden (Grant No. RFR‐939378) funded the study. The funding bodies had no input into the design, conduct, or reporting of the results.

## CONFLICT OF INTEREST STATEMENT

The authors have no conflicts of interest to declare.

## PEER REVIEW

The peer review history for this article is available at https://www.webofscience.com/api/gateway/wos/peer‐review/10.1111/jan.16340.

## Supporting information


Data S1.

## Data Availability

The datasets generated and analysed during the current study are not publicly available, as per the Regional Ethical Review Board's guidelines to protect the privacy of the participants but are available from the corresponding author on reasonable request.
