# Supplementary material for: Bracing for the next wave: A critical incident study of frontline decision‐making, adaptation and learning in ambulance care during COVID‐19
Source: J Adv Nurs. 2024 Jul 17;81(9):5442–57. doi: 10.1111/jan.16340 (PMC12371785; doi:10.1111/jan.16340)
Supplement: Supplementary file 1 — Data S1. [file JAN-81-5442-s001.docx]

Bracing for the next wave: A critical incident study of frontline decision-making, adaptation, and learning in ambulance care during COVID-19

Consolidated criteria for reporting qualitative studies (COREQ): 32-item checklist

Developed from:

Tong A, Sainsbury P, Craig J. Consolidated criteria for reporting qualitative research (COREQ): a

32 item checklist for interviews and focus groups. International Journal for Quality in Health Care.

2007. Volume 19, Number 6: pp. 349 – 357

| No Item | Guide question / description | Reported on page # |
| --- | --- | --- |
| **Domain 1: Research team**  **and reflexivity** |  |  |
| *Personal Characteristics* |  |  |
| 1. Interviewer/ Facilitator | Which author/s conducted the data collection? | The data collection by online surveys was performed by the first author. |
| 1. Credentials | What were the researcher’s credentials? E.g. PhD, MD | The researchers were one PhD student and four PhDs. See Title page |
| 1. Occupation | What was their occupation at the time of the study? | The researchers were two PhDs working at Linnaeus University, one two PhD working at Region Sörmland and one PhD student at Linnaeus University, working at the ambulance service in Region Kalmar County  See Title page (affiliations) |
| 1. Gender | Was the researcher male or female? | The research group consisted of four females and one male. The first author is female. |
| 1. Experience and training | What experience or training did the researcher have? | The first author is a PhD student with supervision by the co-authors who had experience in this method.  Potential author bias was handled by review of memos and peer debriefing during the analysis process.  See Strengths and limitations p. 3-36 |
| *Relationship with participants* |  |  |
| 1. Relationship established | Was a relationship established prior to study commencement? | Researchers established contact with top managers and heads of departments. Informants did not have any relationship with the researchers prior to the study.  See Method, Recruitment and participants p.10 |
| 1. Participant knowledge of the interviewer | What did the participants know about the researcher? e.g. personal goals, reasons for doing the research | Informants were introduced to the research by the heads of departments and did not have contact with the researchers prior to data collection. See Method, Recruitment and participants p.10 |
| 1. Interviewer characteristics | What characteristics were reported about the interviewer/facilitator? e.g. Bias, assumptions, reasons and interests in the research topic | Basic information was reported about the researcher.  See Method, Recruitment and participants p.10 and Strengths and limitations p. 35-36 |
| **Domain 2: study design** |  |  |
| *Theoretical framework* |  |  |
| 1. Methodological orientation and Theory | What methodological orientation was stated to underpin the study? e.g. grounded theory,  discourse analysis, ethnography, phenomenology, content analysis | Critical Incident Technique and Interpretive description  See Methods, Design p.7-8 |
| *Participant selection* |  |  |
| 1. Sampling | How were participants selected? e.g. purposive, convenience, consecutive, snowball | Purposive sampling  See Methods, Recruitment of participants p.10 |
| 1. Method of approach | How were participants approached? e.g. face-to-face, telephone, mail, email | See Methods, Recruitment of participants p.10 |
| 1. Sample size | How many participants were in the study? | In total, 28 participants.  See Methods, recruitment and participants and data collection p.10-11 and Table 1 p.11 |
| 1. Non-participation | How many people refused to participate or dropped out? Reasons? | No participants refused or dropped out |
| *Setting* |  |  |
| 1. Setting of data collection | Where was the data collected? e.g. home, clinic, workplace | The questionnaires were filled out online on the participant’s choice of location.  See Methods p.11-12 |
| 1. Presence of non-participants | Was anyone else present besides the participants and researchers? | No |
| 1. Description of sample | What are the important characteristics of the sample? e.g. demographic data, date | The demographics of the sample is reported in Methods p.10-11 and Table 1 p.11 |
| *Data collection* |  |  |
| 1. Interview guide | Were questions, prompts, guides provided by the authors? Was it pilot tested? | The questionnaire was pilot tested  Methods p.12 |
| 1. Repeat interviews | Were repeat interviews carried out? If yes, how many? | No |
| 1. Audio/visual recording | Did the research use audio or visual recording to collect the data? | No |
| 1. Field notes | Were field notes made during and/or after the interview or focus group? | No |
| 1. Duration | What was the duration of the interviews or focus group? |  |
| 1. Data saturation | Was data saturation discussed? | Thematic saturation was addressed. See Strengths and limitations p.36.  Data saturation in itself is not applicable to the methodology of this study. |
| 1. Transcripts returned | Were transcripts returned to participants for comment and/or correction? | No |
| **Domain 3: analysis and findings** |  |  |
| *Data analysis* |  |  |
| 1. Number of data coders | How many data coders coded the data? | The main author coded the data while the co-authors contributed to validation of coding |
| 1. Description of the coding tree | Did authors provide a description of the coding tree? | No |
| 1. Derivation of themes | Were themes identified in advance or derived from the data? | Derived from data as the analysis was inductive  See Methods, Data analysis p.13-15 |
| 1. Software | What software, if applicable, was used to manage the data? | Google Forms, Microsoft Excel and Microsoft Word |
| 1. Participant checking | Did participants provide feedback on the findings? | No |
| *Reporting* |  |  |
| 1. Quotations presented | Were participant quotations presented to illustrate the themes / findings? Was each quotation identified? e.g. participant number | Yes. Each quotation was identified with participant´s number.  See Result section – for example p.20 |
| 1. Data and findings consistent | Was there consistency between the data presented and the findings? | Consistency between data and the findings is presented with quotations and examples from the analysis process.  Credibility is discussed in Strengths and Limitations p.35-36 |
| 1. Clarity of major themes | Were major themes clearly presented in the findings? | Yes, see Table 4 and Results section p.17-19.  And Figure 2 p.19 |
| 1. Clarity of minor themes | Is there a description of diverse cases or discussion of minor themes? | Yes  See Results section, Table 4 p.18 |
